# Fine Particle Sources and Cardiorespiratory Morbidity: An Application of Chemical Mass Balance and Factor Analytical Source-Apportionment Methods

**DOI:** 10.1289/ehp.10873

**Published:** 2008-01-14

**Authors:** Jeremy A. Sarnat, Amit Marmur, Mitchel Klein, Eugene Kim, Armistead G. Russell, Stefanie E. Sarnat, James A. Mulholland, Philip K. Hopke, Paige E. Tolbert

**Affiliations:** 1 Department of Environmental and Occupational Health, Emory University, Atlanta, Georgia, USA; 2 Department of Civil and Environmental Engineering, Georgia Institute of Technology, Atlanta, Georgia, USA; 3 California Air Resources Board, Sacramento, California, USA; 4 Department of Chemical Engineering, Clarkson University, Potsdam, New York, USA

**Keywords:** acute, Atlanta, cardiovascular, chemical mass balance, emergency department visits, fine particulate matter, positive matrix factorization, respiratory, source apportionment, tracer

## Abstract

**Background:**

Interest in the health effects of particulate matter (PM) has focused on identifying sources of PM, including biomass burning, power plants, and gasoline and diesel emissions that may be associated with adverse health risks. Few epidemiologic studies, however, have included source-apportionment estimates in their examinations of PM health effects. We analyzed a time-series of chemically speciated PM measurements in Atlanta, Georgia, and conducted an epidemiologic analysis using data from three distinct source-apportionment methods.

**Objective:**

The key objective of this analysis was to compare epidemiologic findings generated using both factor analysis and mass balance source-apportionment methods.

**Methods:**

We analyzed data collected between November 1998 and December 2002 using positive-matrix factorization (PMF), modified chemical mass balance (CMB-LGO), and a tracer approach. Emergency department (ED) visits for a combined cardiovascular (CVD) and respiratory disease (RD) group were assessed as end points. We estimated the risk ratio (RR) associated with same day PM concentrations using Poisson generalized linear models.

**Results:**

There were significant, positive associations between same-day PM_2.5_ (PM with aero-dynamic diameter ≤ 2.5 μm) concentrations attributed to mobile sources (RR range, 1.018–1.025) and biomass combustion, primarily prescribed forest burning and residential wood combustion, (RR range, 1.024–1.033) source categories and CVD-related ED visits. Associations between the source categories and RD visits were not significant for all models except sulfate-rich secondary PM_2.5_ (RR range, 1.012–1.020). Generally, the epidemiologic results were robust to the selection of source-apportionment method, with strong agreement between the RR estimates from the PMF and CMB-LGO models, as well as with results from models using single-species tracers as surrogates of the source-apportioned PM_2.5_ values.

**Conclusions:**

Despite differences among the source-apportionment methods, these findings suggest that modeled source-apportioned data can produce robust estimates of acute health risk. In Atlanta, there were consistent associations across methods between PM_2.5_ from mobile sources and biomass burning with both cardiovascular and respiratory ED visits, and between sulfate-rich secondary PM_2.5_ with respiratory visits.

Recent interest in the health effects of particulate matter (PM) has focused on identifying sources of PM that pose the greatest health risks. Because it is likely that not all PM is equally toxic, epidemiologic models that incorporate source-resolved PM may provide a step toward targeting the most important causal agents and refining traditional mass-based PM standards. Quantifying health risks associated with sources such as biomass burning, power plants, gasoline and diesel emissions, rather than individual pollutants, may also capture complex multipollutant interactions that more accurately reflect the etiologic relationships between PM and adverse health. Few epidemiologic studies, however, have included source-apportionment data in their examinations of PM health effects (Ito et al. 2006; Laden et al. 2000; Mar et al. 2000, 2006; Ozkaynak and Thurston 1987; Schreuder et al. 2006). The limited application of source apportionment may be attributable partly to uncertainties regarding optimal methods for conducting PM source apportionment, as well as the lack of suitable air quality data for analysis.

Source-apportionment methods used in previous studies have generally relied on factor analytic approaches. For example, Laden et al. (2000) grouped elemental PM concentrations from six U.S. cities into a small numbers of categories, or “factors” (Laden et al. 2000). Significant associations were found between mortality and the traffic and coal combustion factors, with the largest effect size for the traffic factor. No significant associations were observed for the oil and soil factors.

A series of recent analyses examined the associations between source-resolved PM estimates and mortality in Washington, DC, and Phoenix, Arizona, using several different multivariate factor analytic methods in each city (Hopke et al. 2006; Ito et al. 2006; Mar et al. 2006; Thurston et al. 2005). In these analyses, source apportionment was conducted on samples collected twice a week, using absolute principal components analysis (PCA), UNMIX (a multivariate receptor model), and positive matrix factorization (PMF). Results showed that variability among the methods was small when compared with overall source-apportionment model uncertainty, and suggested that these apportionment methods may be useful in discerning source-specific health effects. The authors note the relatively limited sample size for these data sets and their inability to robustly identify certain source categories (e.g., specific mobile source types). Questions also remain concerning the generalizability of these findings to other locations with different aerosol compositions, the marginal benefit of using source-apportioned data over single-species tracers, and whether analyses using other source-apportionment methods, notably chemical mass balance (CMB), will show the same pattern of agreement.

Here we present and compare results from epidemiologic analyses of emergency department (ED) visits and source-resolved PM_2.5_ (PM with aerodynamic diameter ≤ 2.5 μm; fine PM) obtained using PMF, modified CMB, and a single-species tracer approach. This analysis is the first to compare epidemiologic findings generated using both factor analysis and mass balance source-apportionment methods. The data used in this analysis were collected in Atlanta, Georgia, a unique location for conducting this type of health-effects study given the existence of an extensive time-series of daily speciated PM_2.5_ measurements and corresponding hospital records. These data have been previously characterized in several source-apportionment and epidemiologic analyses (Kim et al. 2004; Marmur et al. 2005; Metzger et al. 2004; Peel et al. 2005). We compare results across methods and assess the robustness of health risk estimates for cardiopulmonary ED visits. The implications of using one or several methods for understanding the sources of PM_2.5_-mediated health risks are also addressed.

## Methods

Source-apportionment methods, including PMF, an extended CMB method, and single-species tracers, were applied to PM_2.5_ concentrations collected daily at an urban Atlanta site, Jefferson Street, between November 1998 and December 2002. The Jefferson Street site is located 4 km northwest of downtown and has served as a primary measurement site for the Southeastern Aerosol Research and Characterization Study (SEARCH), the Aerosol Research Inhalation Epidemiology Study (ARIES), and several ongoing epidemiologic studies as part of the extensive Studies of Particles and Health in Atlanta (SOPHIA) (Hansen et al. 2006; Metzger et al. 2004; Peel et al. 2005; Wade et al. 2006). Detailed particulate speciation was conducted to obtain particulate ionic, trace metals, and temperature-resolved carbonaceous concentrations, via ion chromatography, X-ray fluorescence, thermal-optical reflectance (TOR), respectively. The source-resolved PM_2.5_ was subsequently used in daily time-series analyses to estimate the risk ratio (RR) of respiratory disease (RD) and cardiovascular disease (CVD) ED visits.

### Source apportionment of the Atlanta aerosol

Several source-apportionment studies have been conducted on speciated PM_2.5_ data collected at Jefferson Street. For the current analyses, PM_2.5_ source apportionment was conducted using PMF with temperature-resolved carbon fractions (Kim et al. 2004); an extended CMB approach using the Lipschitz global optimizer (LGO) program, CMB-LGO (Marmur et al. 2005); and single-species source-indicative tracers. The PMF and CMB-LGO analyses on the current data set were conducted, independent of each other, by researchers from Clarkson University and the Georgia Institute of Technology, respectively. Complete descriptions of the specific source-apportionment methods have been published elsewhere (Kim et al. 2004, 2003a, 2003b; Marmur et al. 2005), but brief summaries for each method are presented below.

#### PMF

PMF is a factor analytic method that distinguishes correlation patterns among speciated PM_2.5_ measurements in a given location. As such, it does not rely on *a priori* knowledge concerning chemical profiles of sources to generate source contribution estimates. An often-noted limitation of using factor analysis methods is the inability to link observed factors in the analysis directly with actual sources. Because these methods are based on statistical patterns of correlations, rather than empirical chemical source profiles, naming the factors as specific sources is somewhat subjective.

#### CMB-LGO

CMB receptor models are a common tool for apportioning ambient levels of pollutants among the major contributing sources. CMB combines the chemical and physical characteristics of particles measured at sources and receptors to quantify the source contributions to the receptor. The quantification is based on the solution to a set of linear equations that express each receptor’s ambient chemical concentration as a linear sum of products of source-profile abundances and source contributions. In the enhanced CMB-LGO model, source-indicative sulfur dioxide/PM_2.5_, carbon monoxide/PM_2.5_, and nitrogen oxides/PM_2.5_ ratios are used as constraints, in addition to the commonly used particulate-phase source profiles. A limitation of CMB approaches is the assumption that profiles characterized at the source remain unchanged between source and receptor. For this comparison, both estimated source contributions from CMB-LGO and factor contributions from PMF will be referred to as “source categories.”

#### Tracer method

Species that are characteristic of a given source profile and present in samples above their respective limits of detection may, in some cases, serve as suitable tracers of that source. Several source-indicative tracers were selected *a priori*, based on results from previously published studies (Koutrakis et al. 1992; Laden et al. 2000). For the current analyses, silicon was used as a tracer for soil, potassium for biomass or wood burning, zinc as a tracer for gasoline vehicles, elemental carbon (EC) for diesel sources, organic carbon (OC) for the CMB-LGO source “other OC,” sulfate ion (SO_4_^2−^) for sulfate-dominated secondary aerosol, nitrate ion (NO_3_^−^) for nitrate-dominated secondary aerosol, and selenium for primary emissions from coal-fired power plants (Reddy et al. 2005). None of these elements should be considered unique to a given source.

To facilitate comparisons within and among the methods, most analyses were restricted to days when estimates for all source categories were available. Over the time period analyzed, a total of 1,018 daily measurements were included in the analyses [492 measurements during the cool season (October 15–April 14); 526 measurements during the warm season (April 15–October 14)]. Sensitivity analyses conducted comparing the restricted and complete data sets and their corresponding epidemiologic results showed little difference in the RR estimates and resulting interpretation.

### Emergency department data

Information on individual-level ED visits was collected via electronic billing data from acute care hospitals serving the 20-county Atlanta metropolitan area. For the period November 1998 through December 2002, a subset of the ongoing SOPHIA study period, 27 of 42 hospitals serving the area were able to provide useable electronic data. We categorized ED visits into a combined RD case group using the following primary *International Classification of Disease, 9th Revision* (ICD-9; World Health Organization 1975) diagnostic codes: asthma (493, 786.09), chronic obstructive pulmonary disease (491, 492, 496), upper respiratory infection (460–466, 477), and pneumonia (480–486). A combined CVD group was also created that combined the following primary ICD-9 codes: ischemic heart disease (410–414), cardiac dysrhythmias (427), congestive heart failure (428), and peripheral vascular and cerebrovascular disease (433–437, 440, 443–444, 451–453). ED visits for each outcome group were aggregated by day for use in epidemiologic analyses. Repeat visits within a day by a specific patient were counted as a single visit.

### Data analysis

#### Source impact comparisons

We compared source impacts within and between source-apportionment methods. Pollutant data were non-normally distributed, so we used Spearman’s correlation coefficients. Many of the analyses were conducted using seasonally stratified data, given the differences in pollutant concentrations, distribution, and meteorology occurring in warm compared with cool seasons.

#### Epidemiologic analyses

We estimated the relative risk of daily RD and CVD ED visits associated with 24-hr integrated source impacts using Poisson generalized linear models (McCullagh and Nelder 1989). These analyses are similar to those used in our previous analyses of Atlanta data (Metzger et al. 2004; Peel et al. 2005). The basic form of the model is


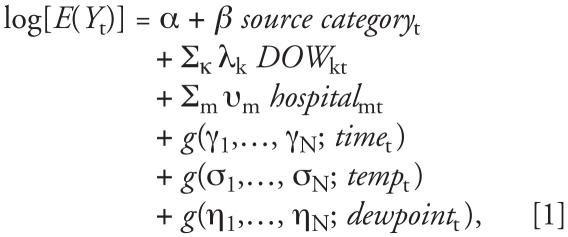


where *Y*_t_ is the count of ED visits on day *t* for the outcome of interest. The model also included indicator variables for day of week and holidays (*DOW*), and hospital indicator variables (*hospital* ) to account for the entry and exit of hospitals into and from the database during the study period. Long-term trends in case presentation rates (*time*) were controlled using semiparametric cubic splines, *g*(γ_1_,…, γ*_N_; x*) with monthly knots in both CVD and RD models. Cubic splines were used to control mean temperature (*temp*) and mean dewpoint temperature (*dewpoint*), with knots placed at the 25th and 75th percentiles. The first and second derivatives of *g*(*x*) were continuous allowing time trends and meteorology to be modeled as smooth functions. Variance estimates were scaled to account for Poisson overdispersion. In this study, we calculated all RRs for an increase of the approximate interquartile range (IQR) of the source category of interest. Relationships were examined with 8 of the 9 CMB sources and 10 of the 11 PMF factors. Relationships with the CMB ammonium bisulfate source and the PMF bus and highway factor were not obtained because of instability resulting from the large number of days in which there were no estimated source contributions from these categories.

In our previous analyses (Metzger et al. 2004; Peel et al. 2005), we observed associations for both cardiovascular and respiratory ED visits with same-day levels of total PM_2.5_. Because an objective of the current analysis was to disaggregate the source-specific health effects associated with total PM_2.5_, for each exposure variable (*source category*) in this analysis, the zero-day lag was chosen as the *a priori* exposure window. Secondary analyses also included models stratified by warm (April 15–October 14) and cool (October 15–April 14) seasons.

## Results

The CMB-LGO and PMF analyses quantified impacts from 9 sources and 11 factors, respectively, for the Atlanta PM_2.5_ concentrations (Table 1). Complete summary statistics for the measured PM_2.5_ concentrations and source categories are presented in Table 2. Six comparable source categories—gasoline vehicles, diesel vehicles, biomass burning or wood smoke, soil, sulfate-rich secondary aerosols, and nitrate-rich secondary aerosols—were identified by both methods. Despite the similar category names and the fact that many of the source categories are driven by similar species, differences exist in the chemical profiles used by each method [Supplemental Material, Table 1 (online at http://www.ehponline.org/members/2008/10873/suppl.pdf)]. To assist in the identification of the factors as source types for PMF, the explained variation provides an indication of which sources are most responsible for explaining the variation in each particular chemical species used in the PMF analysis. A description of explained variation and the results for these data are presented in the Supplemental Material [Table 2 (online at http://www.ehponline.org/members/2008/10873/suppl.pdf)].

**Figure 1:**
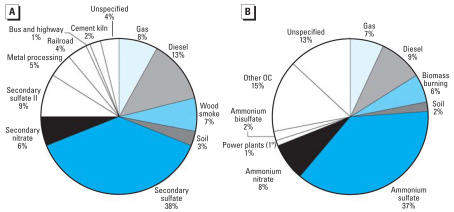
Fractional contribution to PM_2.5_ from (*A*) PMF and (*B*) CMB-LGO source categories at Jefferson Street, Atlanta, GA (November 1998–December 2002).

A substantial fraction of the Atlanta PM_2.5_ was identified as sulfate-rich secondary aerosol, comprising approximately 40% of the total PM_2.5_ mass during the study period (Table 2, Figure 1). Both the PMF and CMB-LGO models identified separate diesel and gasoline engine source categories. Both methods indicated that roughly 20% of the PM_2.5_ mass in Atlanta originated from these two mobile source categories, although PMF apportioned a greater fraction of the total PM_2.5_ mass to diesel as compared with CMB-LGO (13 vs. 9%, respectively). There were other differences regarding the identification of specific categories, such as primary PM_2.5_ from coal-fired power plants, identified in CMB-LGO only, and cement, railroad, bus/highway, and metals-processing factors, identified in PMF only.

### Associations within and between source-apportionment methods

Several source categories within each method were correlated with each other, as expected given the strong influence of meteorologic factors on pollutant temporal patterns [Supplemental Material, Table 3a–b (online at http://www.ehponline.org/members/2008/10873/suppl.pdf)]. In general, the PMF factors were more orthogonal (i.e., less correlated) than the CMB-LGO sources, which was expected given that PMF factors are identified in part on the basis of their lack of collinearity. Moderate temporal correlation (i.e., Spearman’s *r* > 0.40–0.60) existed between the gas, diesel, and biomass burning source categories in CMB-LGO. Similarly, moderate correlation existed among the gas, diesel, and wood smoke PMF categories.

Correlations between PMF and CMB-LGO source estimates were generally strong for the six comparable categories that were identified by both methods (Table 3). For the diesel, biomass burning or wood smoke, soil, and sulfate-rich and nitrate-rich secondary aerosol source categories, correlations exceeded 0.80 during both seasons, except for the warm season correlation between PMF and CMB-LGO soil (*r*_S_ = 0.69). The gasoline source category was a notable point of contrast, with only weak to moderate correlations between the methods during both seasons (*r*_S_ = 0.45 and 0.24 in cool and warm seasons, respectively). Mean absolute deviations between values estimated by both sources were generally < 1 μg/m^3^, which for specific source categories represented large relative differences [Supplemental Material, Table 4 (online at http://www.ehponline.org/members/2008/10873/suppl.pdf)]. Differences between methods were typically greatest for gasoline and soil impacts relative to their respective means, with the sulfate- and nitrate-rich secondary aerosols exhibiting the least relative deviation.

Many of the elemental species were also strongly correlated with specific source categories, supporting their *a priori* selection as source tracers (Table 4). This was not true for all tracer species. For example, Zn was strongly correlated with CMB gasoline source contributions (*r*_S_ = 0.96), but only moderately to weakly correlated with the PMF gasoline estimates (*r*_S_ = 0.42). Se was not a strong tracer for power plant source contributions (*r*_S_ ≈ 0.4), likely because of its relatively low signal-to-noise ratio.

### Associations with RD and CVD visits

Thirty-seven of 42 hospitals in the metropolitan Atlanta area provided data on > 4.5 million emergency department visits for the time period included in these analyses. There was an average (± SD) of 324 ± 123 (range, 105–1,061) and 75 ± 17 (range, 32–123) visits per day for RD and CVD visits, respectively. In the restricted data set, same-day increases in total PM_2.5_ (i.e., a 0-day lag) were significantly associated with increases in CVD visits [RR = 1.022; 95% confidence interval (CI), 1.007–1.038], but not RD visits (RR = 1.005; 95% CI, 0.996–1.015). In the nonrestricted data set for this time period, PM_2.5_ was also significantly associated with RD visits (RR = 1.016; 95% CI, 1.007–1.024) for a 0-day lag.

**Figure 2:**
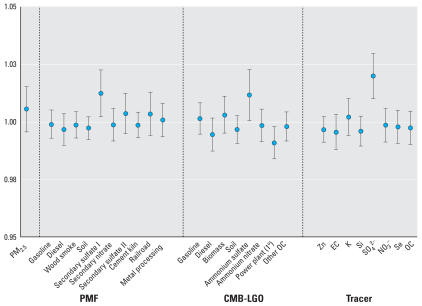
RRs and 95% CIs per IQR increase from same-day lag models for the association of ED visits for all respiratory disease with daily source-apportioned ambient PM_2.5_ (Atlanta, GA, November 1998–December 2002).

Associations between the same-day concentration of the PM_2.5_ source categories and RD visits were consistent with the null for all models using PMF, CMB-LGO, and the tracers, except for the source categories characterizing sulfate-rich PM_2.5_ [Figure 2; Supplemental Material, Table 5 (online at http://www.ehponline.org/members/2008/10873/suppl.pdf)]. An IQR increase in sulfate-rich secondary PM_2.5_, formed mainly from primary power plant precursors, was associated with 1.2–2.0% increases in respiratory visits. In contrast, we found a significant negative association between respiratory visits and primary emissions from coal-fired power plants in CMB-LGO.

**Figure 3:**
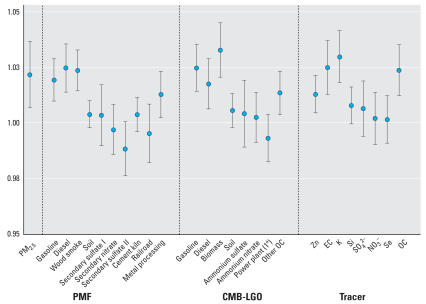
RRs and 95% CIs per IQR increase from same-day lag models for the association of ED visits for all CVD with daily source-apportioned ambient PM_2.5_ (Atlanta, GA, November 1998–December 2002).

Visits to the ED for CVD were significantly and positively associated with CMB-LGO diesel, gas, biomass burning, and other OC source categories [Figure 3, Supplemental Material, Table 5 (online at http://www.ehponline.org/members/2008/10873/suppl.pdf)]. IQR increases in these sources were associated with RR estimates ranging from 1.014 (other OC) to 1.033 (biomass). PMF diesel, gas, wood smoke, and metal processing categories were also significantly and positively associated with CVD visits, with the magnitude of RRs per IQR ranging from 1.013 (metal processing) to 1.025 (diesel). Similarly, EC and K, selected as single-species tracers for the diesel and biomass burning source categories, were also significantly associated with CVD visits.

**Figure 4:**
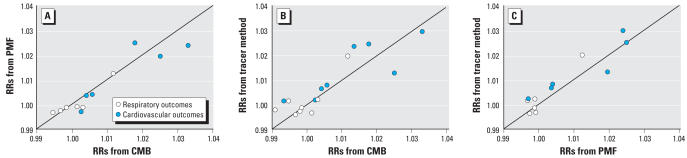
Agreement of estimated RRs (0-day lag) for all RD and CVD between (*A*) CMB and PMF (*R*^2^ = 0.87), (*B*) CMB and single-species tracers (*R*^2^ = 0.76), and (*C*) PMF and single-species tracers (*R*^2^ = 0.87). Observations are taken from the comparable source categories.

Analyses comparing the observed RR estimates for source categories identified by all three methods indicated strong agreement among the three source-apportionment methods. Scatterplots of the RR values from CMB-LGO predicting the RR values from PMF, the RR values from PMF predicting the RR values from the tracer method, and the RR values from CMB-LGO predicting the RR values from the tracer method showed approximate one-to-one associations (Figure 4). Variability in observed RR estimates within the CMB-LGO model output, for example, explained 87% of the corresponding variability in the PMF-based RRs.

There was some indication that associations with CVD outcomes were stronger during the cooler months in Atlanta, whereas associations with respiratory visits were stronger during the warmer months from models stratified between cool and warm months [Supplemental Material, Table 6 (online at http://www.ehponline.org/members/2008/10873/suppl.pdf)]. Pollutant variables found to be associated with ED visits throughout the year were PMF diesel, EC, CMB-LGO gas, Zn, and the three biomass combustion source categories (i.e., CMB-LGO biomass burning, PMF wood smoke, and K). Diesel and gas sources, from both PMF and CMB-LGO, which were not associated with respiratory visits in the overall models, were associated with significant increases in respiratory visits (1.2–2.1% per IQR) during the warm season.

## Discussion

The current results indicate that specific sources are associated with PM_2.5_-related health effects in Atlanta. Additionally, these findings raise new questions concerning the current limitations and resulting value of the source apportionment–epidemiology approach. During this 4-year time-series in Atlanta, there were clear, positive associations between same-day PM_2.5_ concentrations attributed to OC-dominated source categories, such as mobile sources and biomass burning, and ED visits for CVD-related causes. Respiratory visits were also associated with sulfate-rich secondary PM_2.5_ and, during the warmer months, with mobile source–related source impacts. Each of these results was robust to the selection of source-apportionment method, with strong agreement between the RR estimates from the PMF and CMB-LGO models, as well as with results from models using single-species tracers as surrogates of the source-apportioned PM_2.5_ values. Together, these findings indicate a link between these sources or specific chemical components from these sources and acute, adverse cardio-pulmonary responses in Atlanta.

Despite the substantial methodologic differences and sources of uncertainty that differentiate PMF from CMB-LGO, the epidemiologic model results were strikingly similar in direction and magnitude. This analysis was the first, to our knowledge, to compare epidemiologic results incorporating both factor analytical and chemical mass balance source impacts as exposure terms. Several previous studies examined results generated from factor analytic–epidemiologic models exclusively and found generally consistent estimates across methods (Ito et al. 2006; Mar et al. 2006; Schreuder et al. 2006; Thurston et al. 2005). Ito et al. (2006), for example, examined time-series mortality risks associated with PM_2.5_ apportioned impacts from four PCA/UNMIX and two PMF models in Washington, DC. These models identified up to nine factors, with sea salt, oil combustion, and soil having the greatest between-method heterogeneity in factor estimates with secondary sulfate having the least. Analysis of variation showed that variability across source types was approximately 10 times that of variability across methods. In Atlanta, as well, source contribution estimates from PMF and CMB-LGO differed most for soil and least for secondary sulfate, yet showed generally strong agreement.

It is likely that some homogeneity among the risk estimates across methods was attributable to the fact that in Atlanta, many of the PMF factors and CMB-LGO source impacts were driven by similar species (e.g., K with biomass burning or wood smoke, Si with soil, ionic sulfate with secondary sulfate). This fact also explains a key observed finding that the epidemiologic model results using source indicative tracers generated similar RRs to those from the CMB-LGO and PMF models. Together, the results raise the question of whether source-indicative tracers provide an accurate and less analytically intensive alternative to conducting source apportionment. We recommend a cautious approach in using tracers exclusively, however, because many of these species are emitted from numerous sources (e.g., K, used here as a tracer for biomass burning or wood smoke, is also found in soil) and it is likely that the same species may not serve as tracers to the same sources in different locations (e.g., Zn, an additive to lubricating oil, is indicative of gasoline vehicles in a non-heavily industrial city as Atlanta, but is also associated with industrial metals processing in some locations). An alternative approach, therefore, would be to conduct a sensitivity analysis of source-apportionment results, to mathematically identify and quantify the effect of each species on the apportionment process source-indicative tracers for a given local source (Marmur et al. 2006, 2007). Such an analysis is very useful in determining the most influential tracers for each source category in a given location, and can assist in selecting specific tracers to be investigated as part of an epidemiologic analysis. Although correlations between sources and species can provide such initial estimates, these are descriptive rather than conclusive.

Using both factor analytical and chemical mass balance methods in the same analysis may partially compensate for weaknesses in each method. For example, the process of naming factors and linking them with specific sources in PMF analyses is generally subjective. This is not true for CMB-LGO, which only includes sources in a model for which a known source profile exists, though collinearity and exclusion of sources might cause “misplacement” of emissions between the various sources examined. Conversely, CMB processes are constrained by information available for specific source profiles. If information about a specific source profile does not exist, it will not be apportioned as its own source impact within the CMB model. PMF, conversely, empirically examines temporal patterns to identify factors that fit the data set. Typically, modelers will employ several means of optimizing the number of factors selected, including the physical validity of the model (Reff et al. 2007). The mathematical identification of factors within a data set, therefore, may highlight sources that are potentially missing from a CMB model in a given location. Therefore, more certainty is associated with a source category that is identified by both PMF and CMB-LGO.

During this time period in Atlanta, there were consistent associations between PM_2.5_ emitted from both gasoline and diesel sources and CVD and, to a lesser degree, respiratory ED visits. A recent study examining health effects associated with 19 PM_2.5_ components in six California counties also found substantially elevated mortality risks associated with EC and Zn with CVD and respiratory mortalities (Ostro et al. 2007). As such, the Atlanta results contribute to growing observations that mobile source PM_2.5_ is, in part, a key agent responsible for acute cardiovascular outcomes in many locations (Adar et al. 2007; Bigert et al. 2003; Finkelstein et al. 2004; Hoek et al. 2002; Mills et al. 2005; Ostro et al. 2007; Pekkanen et al. 2000; Peters et al. 2001, 2004; Riediker et al. 2004; Salvi et al. 1999; Schwartz 2001; Schwartz et al. 2005; Sioutas et al. 2005). Although the exact biological mechanisms responsible for traffic-mediated cardiovascular effects remain uncertain, several plausible hypotheses have emerged related to autonomic system and inflammatory response pathways (Brook et al. 2004; Donaldson et al. 2001; Pope et al. 2004; Stone and Godleski 1999).

Sulfate-rich secondary PM_2.5_, formed largely from photochemical reactions involving SO_2_ from primary power plant emissions, was the only source category significantly associated with respiratory visits in the primary models. Although other studies have reported similar effects examining particulate sulfate concentrations (Burnett et al. 1997; Fairley 1999; Schwartz et al. 1996), evidence to support this finding is still inconclusive (Reiss et al. 2007). The sulfate finding is interesting given the observed negative association between respiratory visits and primary PM_2.5_ from power plants. Moreover, there was almost no correlation between secondary sulfate aerosol and primary power plant source contributions, reflecting the different atmospheric processes controlling these primary and secondary source impacts. Given the lack of correlation and different composition of these source impacts [Supplemental Material, Table 2 (online at http://www.ehponline.org/members/2008/10873/suppl.pdf)], the difference in the observed health response is not surprising.

The negative association between primary coal-fired power plant emissions and health outcomes should not be viewed as protective. Primary emissions from coal-fired power plants have the most significant impact on the Jefferson Street monitoring site under very specific conditions (i.e., when the wind is blowing from a power plant that is 7 km away, and when the mixed layer is high enough to entrain those emissions). However, the width of the power plant plume beyond 7 km is quite narrow, and a rather small fraction of the sampled area is being affected at that same time. On the other hand, having relatively stronger winds and a higher mixed depth will decrease many other primary emissions.

Associations of PM_2.5_ components and sources with ED visits for CVD were typically stronger during the cooler months, and associations of respiratory visits stronger during the warmer months, which is consistent with findings from our previous Atlanta analyses (Metzger et al. 2004; Peel et al. 2005). The impact of season on specific source impacts may be explained by atmospheric conditions that predominate during specific times of the year. During the cooler months, reduced photochemistry and greater atmospheric stability typically leads to the enhanced impact from local sources of primary PM_2.5_, such as that from gasoline and diesel vehicles. Conversely, photochemistry during the warmer months will enhance the contribution of secondary PM_2.5_ from regional sources, such as sulfate-rich secondary aerosols and secondary organic aerosol. Biomass burning or wood smoke was the sole source category that showed consistent year-round associations that, in the current analyses, were with CVD visits. This may be explained by the fact that, among the sources examined, it is one of the few that has considerable local (e.g., residential or industrial wood burning) and regional contributions (e.g., prescribed agricultural burning) (Marmur et al. 2006; Zheng et al. 2007). Alternatively, these results could reflect seasonal variation in risk factors not associated with air pollution, for which the analyses are not adequately accounting. Respiratory illnesses, for example, are higher in the cold and flu season, which makes potential air pollution effects more difficult to ascertain during the cold season. Heat stress is a risk factor for CVD illnesses and may lead to a reduced ability to detect air pollution effects in the warm season.

In Atlanta, biomass burning, due to prescribed forest management burning in the summer and residential wood burning in the winter, contributed a sizeable fraction of the PM_2.5_. Associations between PM_2.5_ attributed to biomass burning and CVD visits were somewhat unexpected, however. Several previous PM epidemiologic studies examining PM_2.5_ from this source have found little or no effect on CVD outcomes (Ito et al. 2006; Mar et al. 2006; Schreuder et al. 2006), yet fairly consistent associations related to a variety of respiratory end points (Larson and Koenig 1994; Schreuder et al. 2006; Zelikoff et al. 2002). A few studies have noted similar associations that lend plausibility to the Atlanta findings. Ostro et al. (2007) recently presented results showing significant associations between ambient K concentrations and CVD-, but not respiratory-, related mortality in California. Additionally, several controlled experiments have shown relationships between wood smoke inhalation and markers of acute systemic inflammatory response in animal and human models (Barregard et al. 2006; Barrett et al. 2006). Barrett et al. (2006) found increased levels of serum amyloid A, a cardiovascular risk factor, as well as other markers of plasma coagulability in 13 human subjects exposed to PM wood smoke concentrations over a 4-hr duration. Evidence also suggests that pulmonary inflammation, more commonly associated with biomass burning, may precede a systemic autonomic and inflammatory response as a potential step in the biological pathway of PM-mediated cardiovascular health effects (Brook et al. 2002; Gold et al. 2000).

The CVD response associated with PM_2.5_ from biomass burning or wood smoke was similar in significance and lag structure to that attributed to the diesel source category. It is possible that the compounds responsible for the observed mobile source-related CVD effects may also be present in Atlanta biomass emissions, and that meteorology-induced temporal correlations between the sources led to similar observed responses. Indeed, controlled speciation studies of wood smoke and motor vehicles have identified multiple shared particle and gas-phase organic compounds in their emissions (Schauer and Cass 2000; Zheng et al. 2002). A previous analysis of measurement sites in the southeastern United States, including Atlanta, for example, found that both gasoline vehicles and biomass burning jointly contributed to the local distributions of several polycyclic aromatic hydrocarbon concentrations (i.e., indeno[*cd*]pyrene and indeno[*cd*]fluoranthene) (Zheng et al. 2002). Broadly, the observed CVD responses for many of the source categories characterized by high organic content may reflect the presence of shared compounds in the emissions or similar particle attributes, such as oxidation potential, common to organic carbon species. This interpretation may also explain the strong association between the non-source-apportioned total OC concentration and CVD visits.

Multivariate modeling to control for potential confounding is particularly challenging with source-apportionment variables, given the differential uncertainties associated with each source category. In the current analysis, for example, the results showing positive associations with PM_2.5_ from gasoline sources may represent a true association with PM_2.5_ from this source or the gas source may be serving as a surrogate for exposure to PM_2.5_ from a co-varying source, such as PM_2.5_ from diesel, biomass burning, or other, secondary organic aerosol. In this setting, the effects of source variables measured with greater error will be transferred, in part, to those variables with less measurement error (Schwartz and Coull 2003; Zeger et al. 2000). Additionally, multiple sources often contain common pollutant constituents, further complicating the interpretation of parameter estimates obtained from multisource models. Finally, methods for adjusting model parameters based on empirical uncertainty estimates, such as regression calibration, are questionable because each source category is an aggregate of numerous pollutants each with their own error distribution and covariances.

Future analyses using data with greater pollutant resolution, especially in the organic fraction, may elucidate some of the outstanding issues raised in the current analysis. Organic fingerprints of specific sources exist for a subset of these data (Zheng et al. 2000, 2007) and will contribute toward our ability to disaggregate OC-dominated source impacts. Planned analyses involving individual ED case groups will be helpful in disaggregating nuances in health response that we found when using the aggregated health out-come categories. Specific PM_2.5_ sources or components, for example, may be associated with certain health responses but not others (e.g., congestive heart failure compared with ischemic heart disease) due to etiologic differences among these subcategories. Despite the limitations of current source-apportionment methodology, these findings collectively suggest that one can use variables describing sources in epidemiologic models to obtain estimates of PM_2.5_ acute health effects. Moreover, combining multiple source-apportionment methods adds information that compensates for limitations of relying on any single method.

## Figures and Tables

**Table 1:** Source-apportionment category names by method.

PMF factors	CMB-LGO sources	Tracer
Gasoline	Gasoline	PM_2.5_ Zn
Diesel	Diesel	PM_2.5_ EC
Wood smoke	Biomass burning	PM_2.5_ K
Soil	Soil	PM_2.5_ Si
Secondary sulfate I	Ammonium sulfate	PM_2.5_ sulfate
Secondary nitrate	Ammonium nitrate	PM_2.5_ nitrate
Secondary sulfate II	—	—
Metal processing	—	—
Railroad	—	—
Bus and highway	—	—
Cement kiln	—	—
—	Power plants (1°)	PM_2.5_ Se
—	Other OC	PM_2.5_ OC
—	Ammonium bisulfate	

Comparable source categories are listed on the same row.

**Table 2:** Mean, median, and selected percentiles (10th, 90th) of daily PM_2.5_ source contributions by season from the ARIES monitoring station at Jefferson Street, Atlanta, GA (November 1998–December 2002).

	Cool season (*n* = 492 days)				Warm season (*n* = 526 days)			
	Mean	10th	Median	90th	Mean	10th	Median	90th
Total PM_2.5_	15.8	7.5	14.3	25.5	18.2	9.1	17.0	29.0
PMF diesel	2.6	0.5	1.8	6.3	1.9	0.3	1.5	3.9
CMB diesel	1.5	0.4	1.2	3.3	1.4	0.5	1.2	2.6
PM_2.5_ EC	1.7	0.6	1.4	3.3	1.4	0.6	1.3	2.5
PMF gasoline	1.7	0.3	1.2	3.6	1.1	0.3	0.9	2.3
CMB gasoline	1.5	0.5	1.3	2.9	1.0	0.3	0.9	1.9
PM_2.5_ Zn (ng/m^3^)	15.7	4.6	11.7	30.2	10.9	3.3	8.5	20.2
PMF wood smoke	1.6	0.5	1.3	2.8	0.8	0.1	0.7	1.4
CMB biomass burning	1.1	0.5	1.0	2.0	0.9	0.4	0.8	1.7
PM_2.5_ K (ng/m^3^)	63.0	24.3	53.9	114.2	52.7	23.2	43.3	93.5
PMF soil	0.3	0.0	0.3	0.7	0.8	0.2	0.6	1.3
CMB soil	0.2	0.0	0.1	0.4	0.4	0.1	0.3	0.7
PM_2.5_ Si (ng/m^3^)	67.7	24.3	54.1	123.5	110.9	32.9	89.0	186.3
PMF secondary sulfate I	4.4	1.4	3.7	8.4	8.7	3.0	7.6	16.4
CMB ammonium sulfate	4.2	1.6	3.7	7.5	8.2	3.3	7.3	14.3
PM_2.5_ SO_4_^2−^	3.4	1.5	0.6	5.8	6.0	2.3	5.2	10.8
PMF secondary nitrate	1.4	0.4	1.2	2.6	0.6	0.2	0.5	1.1
CMB ammonium nitrate	2.0	0.6	1.7	3.6	0.9	0.4	0.7	1.6
PM_2.5_ NO_3_^−^	1.4	0.5	1.2	2.6	0.7	0.3	2.9	1.2
CMB power plants^a^	0.1	0.0	0.1	0.3	0.1	0.0	0.1	0.3
PM_2.5_ Se (ng/m^3^)	1.4	0.4	1.1	3.0	1.2	0.4	0.9	2.7
CMB other OC	2.6	0.9	2.1	4.8	2.5	1.1	2.3	4.2
PM_2.5_ OC	4.6	1.9	3.9	8.0	4.0	2.1	3.7	6.4
CMB ammonium bisulfate	0.4	0.0	0.0	1.5	0.4	0.0	0.0	1.1
PMF secondary sulfate II	1.4	0.0	1.3	2.6	1.7	0.4	1.5	3.2
PMF cement kiln	0.4	0.1	0.3	0.8	0.4	0.1	0.3	0.8
PMF bus and highway	0.1	0.0	0.0	0.3	0.1	0.0	0.0	0.2
PMF railroad	0.5	0.1	0.5	0.9	0.7	0.2	0.7	1.3
PMF metal processing	0.8	0.1	0.6	1.8	0.7	0.1	0.6	1.5

Values reported in milligrams per cubic meter unless otherwise specified. Cool season is October 15–April 14; warm season is April 15–October 14. Secondary sulfate II contains a greater organic fraction than secondary sulfate I, which is mainly sulfate and ammonia.

a Reflects impacts from primary power plant emissions solely.

**Table 3:** Spearman’s correlation coefficient between PMF and CMB-LGO source categories during the cool season (10/15–4/14) and warm season (4/15–10/14) (November 1998–December 2002).

	PMF										
CMB	Gas	Diesel	Wood smoke	Soil	Secondary sulfate I	Secondary nitrate	Secondary sulfate II	Metal processing	Railroad	Bus and highway	Cement kiln
Cool season											
Gas	0.45	0.72	0.12	0.61	0.20	0.29	0.00	0.69	–0.15	0.44	0.37
Diesel	0.57	0.88	0.16	0.45	0.20	–0.09	0.04	0.43	0.13	0.46	0.21
Biomass burning	0.23	0.33	0.89	0.28	0.25	–0.21	0.03	0.20	0.10	0.27	0.30
Soil	0.67	0.73	0.29	0.83	0.31	0.14	0.10	0.42	–0.26	0.33	0.25
Ammonium sulfate	0.20	0.31	0.30	0.40	0.85	0.04	0.21	0.16	–0.03	0.00	0.15
Ammonium nitrate	0.13	0.26	–0.03	0.29	0.18	0.89	0.11	0.27	–0.18	0.14	0.15
Power plants (1°)	–0.08	0.01	0.38	0.04	0.00	0.01	0.20	–0.01	0.13	–0.01	0.76
Ammonium bisulfate	–0.13	–0.12	–0.12	–0.08	0.09	0.28	–0.01	–0.05	–0.06	0.02	–0.10
Other OC	0.80	0.76	0.11	0.45	0.16	–0.10	0.16	0.27	–0.01	0.30	0.07
Warm season											
Gas	0.24	0.58	0.06	0.44	0.24	0.31	0.17	0.62	0.02	0.25	0.56
Diesel	0.28	0.81	0.15	0.30	0.34	0.22	0.23	0.31	0.29	0.27	0.46
Biomass burning	0.23	0.34	0.94	0.19	0.04	–0.09	0.14	0.06	–0.01	0.06	0.39
Soil	0.60	0.62	0.49	0.69	0.29	0.16	0.20	0.21	–0.25	0.02	0.44
Ammonium sulfate	0.10	0.40	0.00	0.27	0.96	0.25	0.41	0.21	0.11	–0.03	0.26
Ammonium nitrate	0.15	0.44	–0.05	0.28	0.28	0.87	0.12	0.29	0.05	0.17	0.27
Power plants (1°)	0.11	0.30	0.37	0.28	0.28	0.01	0.19	0.17	0.06	0.11	0.66
Ammonium bisulfate	0.10	0.02	0.07	0.09	0.26	–0.05	0.06	–0.10	–0.16	–0.09	0.00
Other OC	0.61	0.68	0.11	0.30	0.43	–0.04	0.48	0.04	0.06	0.10	0.26

Secondary sulfate II contains a greater organic fraction than secondary sulfate I, which is mainly sulfate and ammonia.

**Table 4:** Spearman’s correlation coefficients between single species tracers, CMB, and PMF by season.

		Cool season correlation with		Warm season correlation with	
Source category	Tracer	CMB	PMF	CMB	PMF
Gas	Zn	0.96	0.42	0.94	0.19
Diesel	EC	0.96	0.95	0.96	0.88
Wood smoke/biomass burning	K	0.99	0.82	0.98	0.62
Soil	Si	0.83	0.82	0.96	0.92
Secondary sulfate I/ammonium sulfate	SO_4_^2−^	0.87	0.97	0.96	0.99
Secondary nitrate/ammonium nitrate	NO_3_^−^	0.94	0.95	0.95	0.92
Power plant	Se	0.39	—	0.50	—
Other OC	OC	0.91	—	0.89	—

Cool season is October 15–April 14; warm season is April 15–October 14.
